# Supplementary figures and images for: DNA replication dynamics during erythrocytic schizogony in the malaria parasites Plasmodium falciparum and Plasmodium knowlesi
Source: PLoS Pathog. 2022 Jun 22;18(6):e1010595. doi: 10.1371/journal.ppat.1010595 (PMC9255763; doi:10.1371/journal.ppat.1010595)

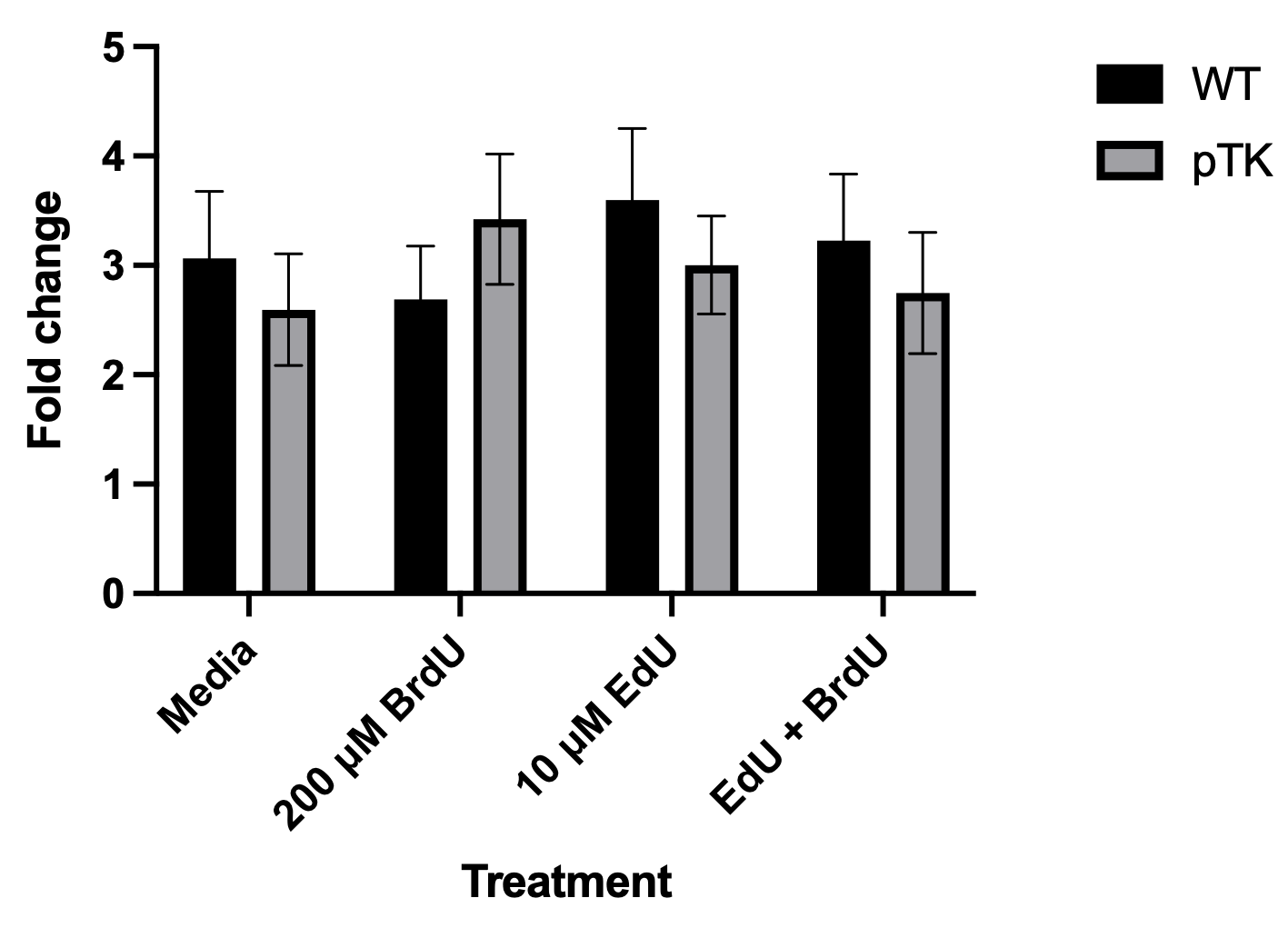

Supplement: S1 Fig — Synchronised parasites of both lines were exposed to either or both modified nucleosides at the levels used in subsequent experiments (10 μM EdU, 200 μM BrdU) for a 6h period covering the majority of S-phase. DNA content was then measured via SYBR-green 1 DNA dye, as previously published [36], in triplicate, and expressed as fold-change in DNA content from the start of the experiment. No significant difference in growth rates was observed in either line, exposed or not exposed to modified nucleosides. (TIF) [file ppat.1010595.s001.tif]

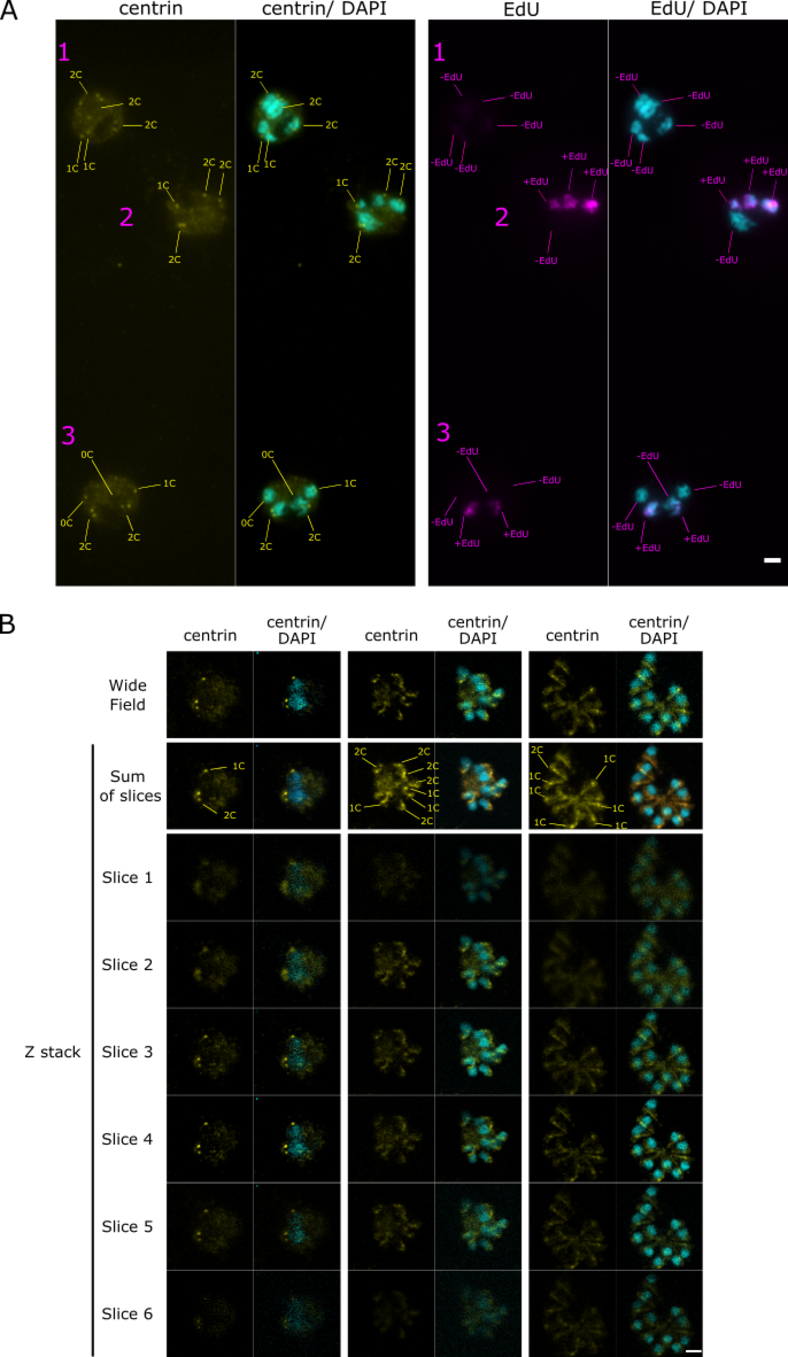

Supplement: S2 Fig — Examples of A) a typical slide, showing 3 parasites classified for their number of nuclear masses, number of centrin foci and presence/absence of EdU staining. B) confocal microscopy versus the wide-field method used throughout this work, showing that confocal yields very similar detection of the number of features per cell. (TIF) [file ppat.1010595.s002.tif]

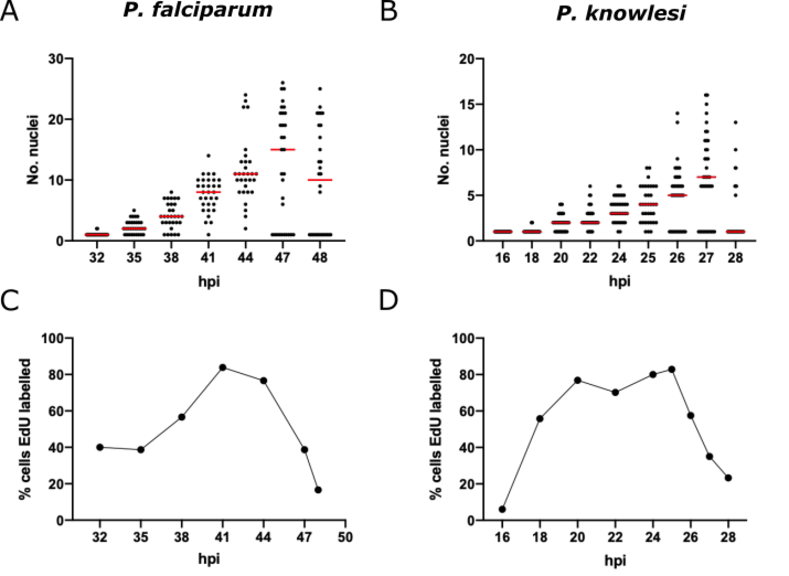

Supplement: S3 Fig — In the early parts of the timecourses, samples were taken every 3h for P. falciparum and every 2h for P. knowlesi, rather than every hour, as in Fig 2. In the final parts of both timecourses, samples were again taken every hour. Final timepoints in both timecourses show reinvasion, i.e. high proportions of 1n cells. A, B: Scatter graphs of nuclear numbers in P. falciparum (A) or P. knowlesi (B), n = 30 cells, medians are shown in red. C, D: Percentage of 30 cells showing some EdU labelling at each timepoint in P. falciparum (C) and P. knowlesi (D). (TIF) [file ppat.1010595.s003.tif]

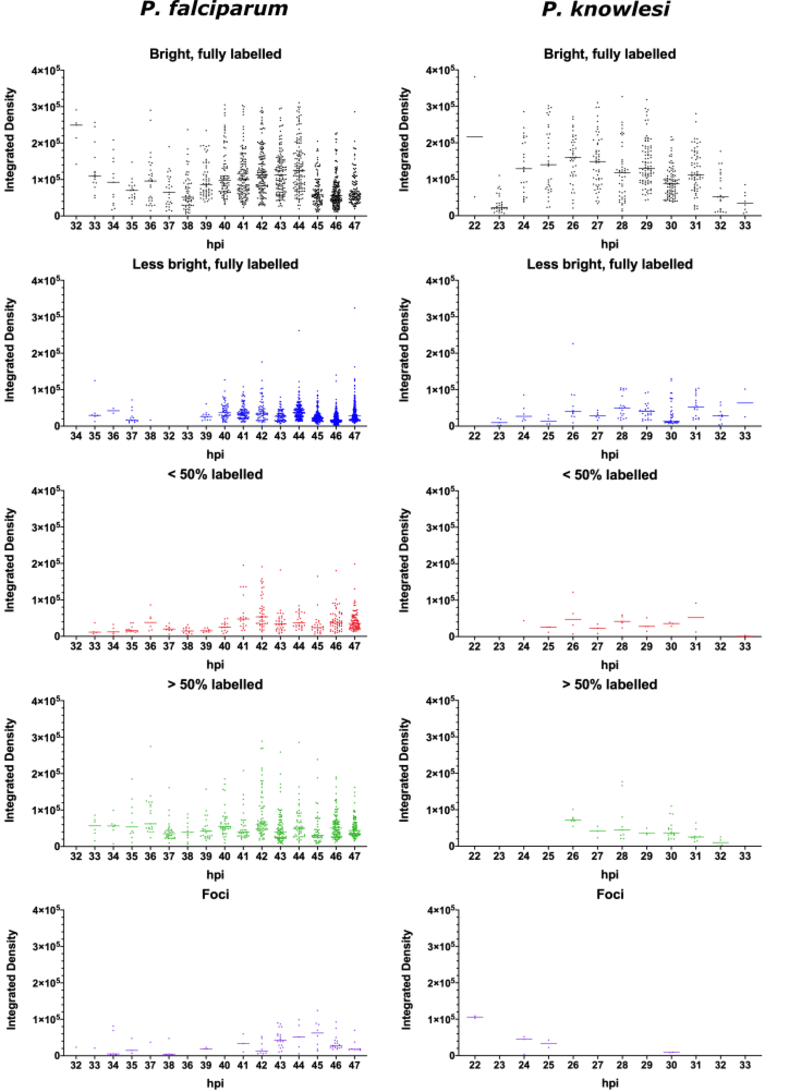

Supplement: S4 Fig — (TIF) [file ppat.1010595.s004.tif]

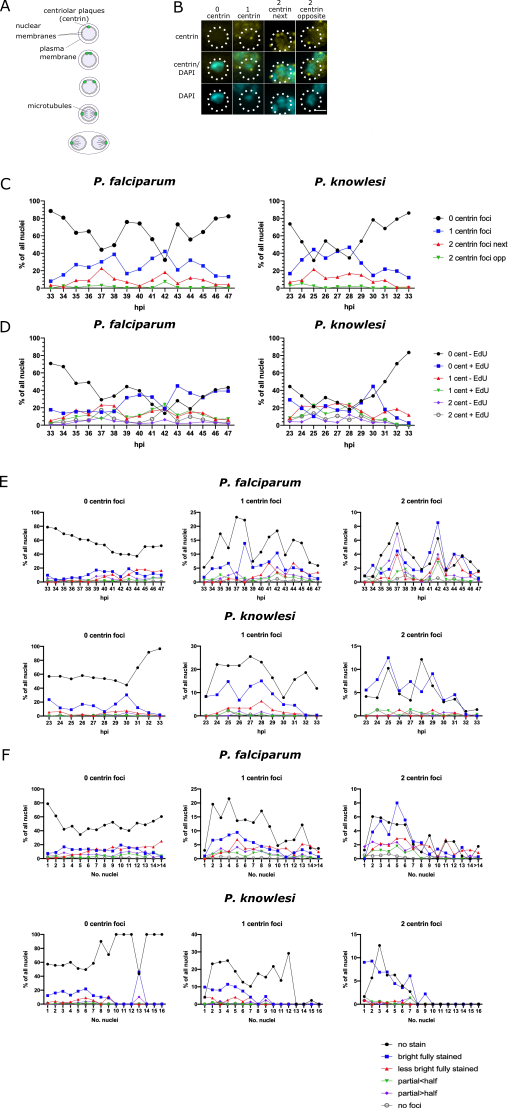

Supplement: S5 Fig — A: Schematic showing the process of karyokinesis that has previously been proposed in Plasmodium, highlighting the role of the centriolar plaque (basis outlined in Gerald et al. [13]). B: Examples of the distinct patterns of centrin staining seen on the highlighted nuclear masses: no foci, a single focus, 2 adjacent foci, 2 opposite foci. P. falciparum are shown, representative of pattern in both species. Scale bar all panels 1μm. C: Percentage of nuclear masses with 0, 1 or 2 centrin foci throughout schizogony, n = 100. D: Percentage of nuclear masses with 0, 1 or 2 centrin foci that also showed or did not show active DNA replication (EdU staining) within the previous 30mins. E: Percentage of nuclear masses with 0, 1 or 2 centrin foci and patterns of intranuclear DNA replication (full, partial, or discrete foci) throughout schizogony. F: Data as in Data as in A, replotted by number of by number of nuclear masses per cell rather than hpi. (PNG) [file ppat.1010595.s005.png]

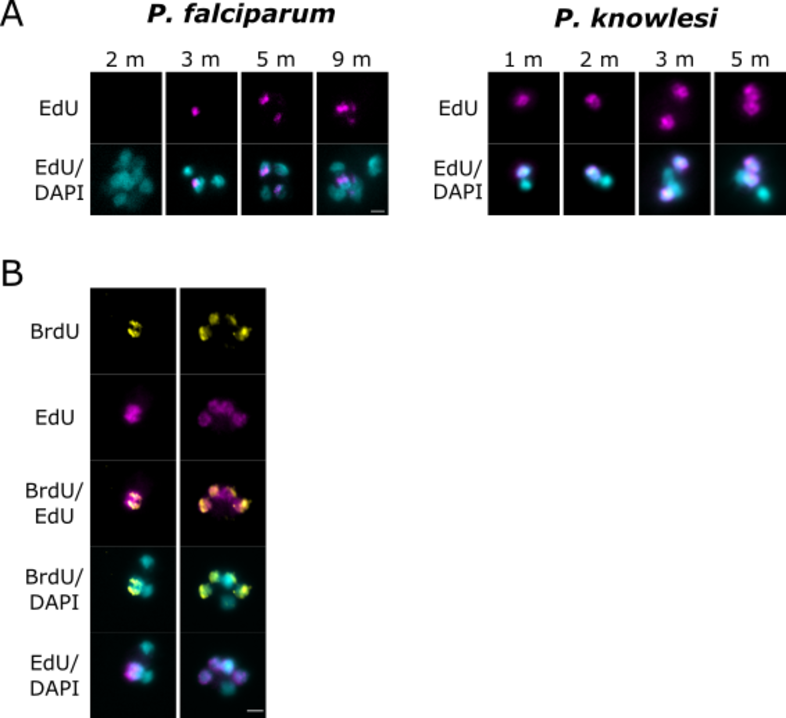

Supplement: S6 Fig — A: In P. falciparum, 3 minutes was detectable (faintly) and 5 minutes gave reasonably bright signal. In P. knowlesi, labelling was clearly detectable within 1 minute. B: Examples of the appearance of stained nuclei when cells are simultaneously labelled with EdU and BrdU. Scale bar all panels 2μm. (TIF) [file ppat.1010595.s006.tif]

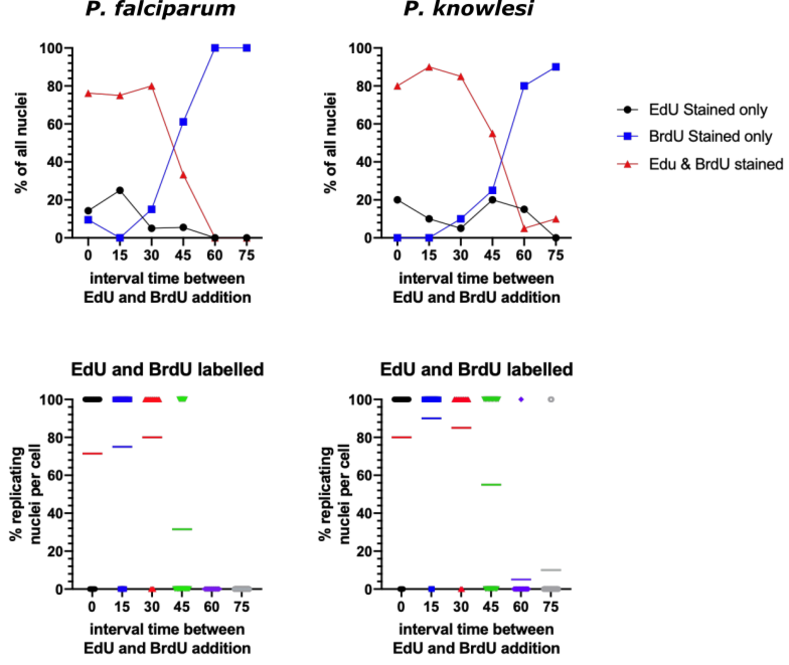

Supplement: S7 Fig — Graphs show the percentage of nuclei in 1n cells that labelled with EdU and BrdU (n = 20). Scatter plots display the same data broken down per-cell, with means shown. (TIF) [file ppat.1010595.s007.tif]

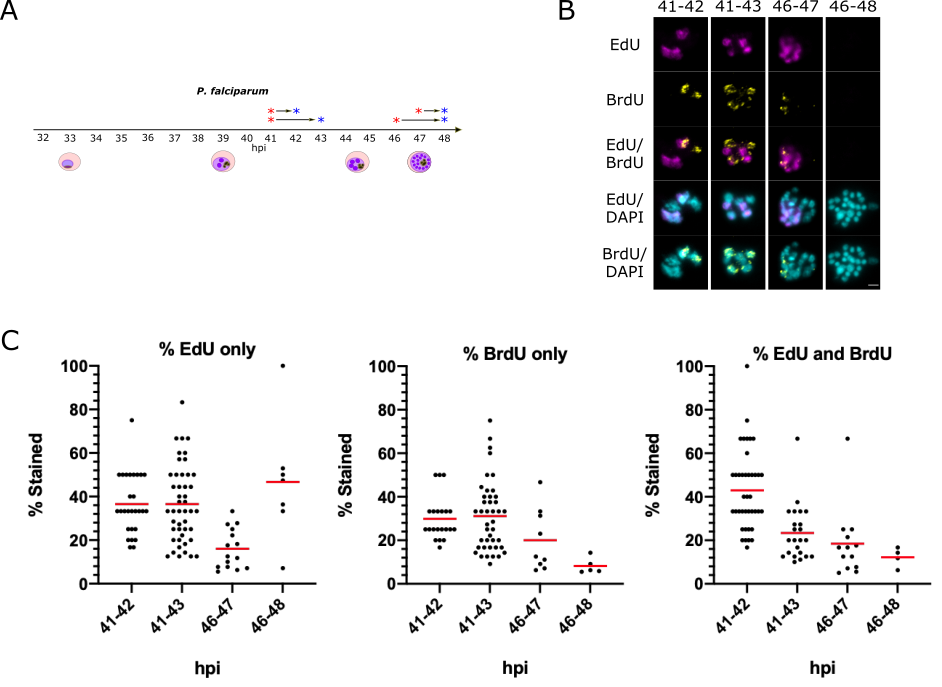

Supplement: S8 Fig — A: Schematic of the timecourses shown in this figure. B: Representative examples of cells across the double-labelled timecourse with 2h intervals for P. falciparum. Scale bar all panels 2μm. B: Percentages of nuclear masses labelled with EdU alone, BrdU alone, or both labels throughout the timecourses shown in (B). (TIF) [file ppat.1010595.s008.tif]

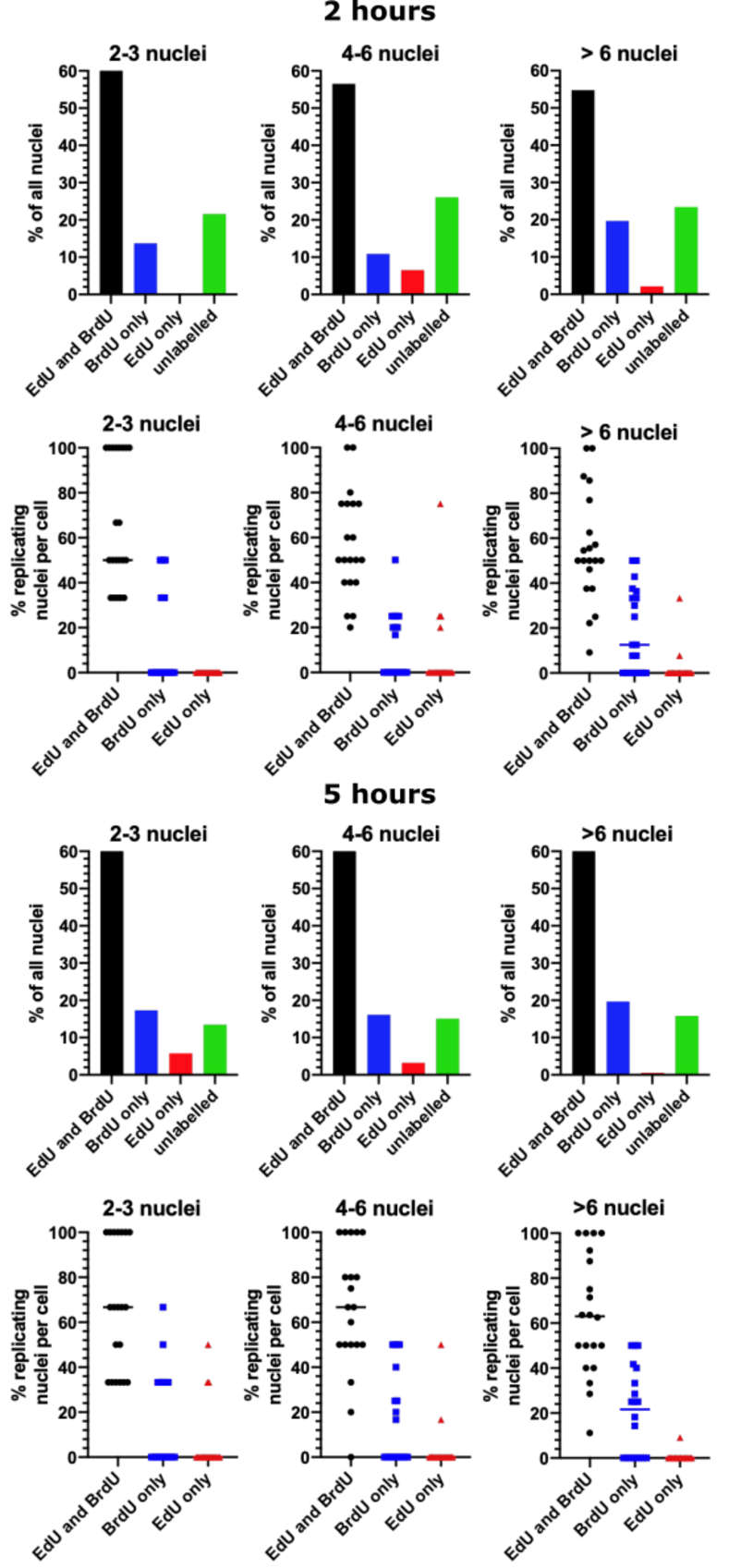

Supplement: S9 Fig — Data are also stratified into cells with 2–3, 4–6, or more than 6 nuclear masses, showing that arrested nuclei are still detected in very young 2-3n schizonts. (TIF) [file ppat.1010595.s009.tif]
