# Supplementary material for: DNA replication dynamics during erythrocytic schizogony in the malaria parasites Plasmodium falciparum and Plasmodium knowlesi
Source: PLoS Pathog. 2022 Jun 22;18(6):e1010595. doi: 10.1371/journal.ppat.1010595 (PMC9255763; doi:10.1371/journal.ppat.1010595)
Supplement: Attachment — Submitted filename: Final rebuttal.docx [file ppat.1010595.s010.docx]

Dear Professor Blackman,

Thank you for the opportunity to revise this manuscript. We have provided a point-by-point response to the reviewers below, and have addressed all of their queries in a substantially revised manuscript. We hope that it will now be acceptable for publication.

Sincerely,

Catherine Merrick

***********************

Reviewer's Responses to Questions

**Part I - Summary**

Reviewer #1

The authors provide an interesting description of replication dynamics throughout erythrocytic schizogony of two Plasmodium species. The amount of data generated is remarkable and the used technology is interesting. However, the study remains descriptive, and the draw conclusion do not extend significantly enough upon the current understanding of replication. The gain of sustainable insight and the ability to generate new hypotheses is a bit too limited to warrant a publication in a journal like PLoS Pathogens.

We appreciate this reviewer’s acknowledgement of the very large amount of data generated here, using a novel technology. We are aware that it is descriptive in nature, but we consider this to be essential groundwork prior to intervention studies (now underway). In fact, the current work generates many newly-testable hypotheses in this regard. For example, how are replication dynamics influenced by glucose limitation, as in calorie-restricted hosts, or by antimalarials such as artemisinins that can damage DNA or antifolates that interfere with nucleotide supply? Then, what are the molecular mechanisms controlling flexible replication dynamics? The final paragraph of our discussion alludes to these questions.

Further, there are some important technical concerns that would have needed to be more thoroughly addressed by the authors to consolidate their data. Lastly the manuscript itself suffers from a difficulty to understand the individual experimental parameters and outcomes. Given some improvements I, however, know that this study can find its place in another journal. In the following I want to outline my criticism in more detail to help the authors to improve the manuscript.

We have addressed the technical concerns below, and tried to make individual experimental parameters clearer in the text and via added schematics. (We note that, by contrast, reviewer #3 found the manuscript very clearly written).

Reviewer #2

In a patient infected with Malaria, Plasmodium parasites replicate in red blood by an atypical cell division mode called schizogony. Multiple rounds of asynchronous DNA replication and nuclear fission generate a multinucleated cell and, specialized cytokinesis named segmentation produces 10 to 30 daughter parasites depending on the Plasmodium species. As McDonald et al. highlighted in their study, the rate and efficiency of parasites replication are fundamentally critical to malaria pathogenesis. In the case of malaria infection by P. falciparum, peripheral parasitemia will determine the severity of the disease. To better understand the molecular mechanisms regulating the number of daughter parasites, the study's authors took a very original and ambitious approach by studying schizogony in two different human Plasmodium parasites, falciparum, and knowlesi, that differ in their cell cycle length and the number of progeny. The authors successfully generated parasite lines that allow de novo DNA replication to be followed by incorporating pulse labels of modified nucleotides. The authors performed different time courses using single EdU pulse labels or sequential pulses of two nucleotides (EdU and BrdU). All experiments readouts were done by light microscopy, except for the determination of the replication fork speed on spreading DNA fibers from cell lysates on glass slides.

Again, we appreciate the reviewer’s acknowledgement of the original and ambitious approach taken here.

Unfortunately, I have significant concerns regarding the experimental robustness of the study, where it appears that for all data presented in the study, there is only a single biological replicate.

It is not really the case that we relied on single biological replicates, because both timecourses shown in Figure 2 (Pf and Pk) were replicated with the addition of 2-label gapped timepoints. These can also be analysed for a single label (as we showed in the original supp Fig 2), and we considered this (as we had noted in the text, now at line 283) to be the most efficient way to leverage the necessity for reproducibility while simultaneously gaining new data. However, we acknowledge the lack of ‘exact’ technical replicates, and therefore we have now generated these, repeating both the Pf and Pk timecourses as shown in Fig 2 and also the S-phase-length experiment shown in what is now Fig 4. These replicates appear as supplementary data (new supp Fig 3 and 7).

In addition, the authors' microscopy technique does not provide the required resolution to resolve two nuclei, especially in the z-axis when they analyze multinucleated with more than four nuclei. Therefore the authors can observe and measure DNA replication per cell but not per nucleus.

We accept that basic light microscopy lacks the resolution of confocal or super-resolution, but it was simply impractical to use more laborious techniques for the many thousands of cells examined here. To generate the data we wished to generate, it was important to look at significant numbers of cells across many timepoints, rather than a small number of cells in great detail. (We acknowledge that this is quite the opposite direction to that taken by other leading workers in the field, who are examining few cells in exquisite detail by super-res and EM. Both approaches can actually give valuable and complementary information.)

In fact, we do not miss a lot of detail at the level required in this study by using basic microscopy. Since the *Plasmodium* cells in our preparations are small and fairly flattened, they exist completely within just a few Z-slices and the gains from confocal microscopy are actually very marginal. We have added evidence to supp figure 2, showing confocal versus single-projection microscopy on a selection of cells, confirming that little detail is missed.

Furthermore, they wrongly interpreted the published data on the MTOC dynamics in Simon et al.'s study, leading them to inappropriately conclude that 50 % of nuclei lack centrin staining, likely representing false negatives. In conclusion, I have raised significant comments that need to be addressed, and numerous statements must be revised based on the limitation of the experimental settings. In addition, I made some minor comments to help the authors identify sections of the study that remain unclear to me.

Concerning MTOC dynamics, we acknowledge that we misinterpreted the text of an early preprint from Simon et al., in extending their findings from 1-n cells to many-n cells. With Dr Guizetti we have since discussed our own preprint versus his now-published paper, and together we agreed on some potential reasons why our results differ: any under-detection of centrin foci is probably due to our use of a commercial antibody. After encountering similar limitations, Dr Guizetti’s group chose to raise their own antibody, together with a *Plasmodium* GFP-tagged line. We have modified the text of our own manuscript accordingly (line 224).

Reviewer #3

 This work offers a detailed and comparative analysis of DNA replication dynamics of two distinct malaria parasite species. P. knowlesi and P. falciparum. It is of particular importance as the first paper for which the replication dynamics of Pk are described, and uses a series of neat approaches to compare the specific replication parameters. This comparison is particularly important as the two parasites have significant differences in cycle length (Pf 2x as long) and AT-content, as well as ultimately producing differing numbers of progeny merozoites. The studies revealed surprising similarities in the single molecule speed of replication as well as inter origin distance, but some clear differences in whole cell replication dynamics – with a surprisingly synchronous onset of S phase and shorter gap phases.
Although descriptive the data presented are of fundamental importance to understanding these processes, and thus I would say they are both highly important for work in the field, and would provide significant interest to the pathogen research community. The paper is very well written and the conclusions are well supported by the data. In the current version, it is slightly unclear as to what biological repeats have been carried out for each experiment, and this is something that will need to be clarified. The other issues raised are relatively minor, although efforts to examine perturbations of these dynamics would add another level of interest.

We appreciate this reviewer’s positive assessment of the strengths of our manuscript, particularly in offering entirely novel data on *P. knowlesi*. We agree that perturbation studies are the next step (now underway), but we believe that waiting to add these would make the paper simply too unwieldy for a single manuscript. We have modified the manuscript re. addition of biological replicates (line 165-67), as also highlighted by reviewer-2.

**Part II – Major Issues: Key Experiments Required for Acceptance**

Reviewer #1: The authors don´t comment sufficiently on the very low mean number of nuclei at the end of schizogony (P.f. 11 and P.k. 6) shown in Fig. 2. This stands in contrast to what has been measured in several studies by e.g. Garg et al. , Reilly et al., Simon et al. and would warrant a more detailed analysis. The observation that number of nuclei is highly variable is not novel as suggested in the abstract.

We appreciate that our median numbers are smaller than previously reported, but the max. number of merozoites reached in our cells is similar to the max. reported in previous studies of *P. falciparum* (28) and *P. knowlesi* (16), suggesting that the biology is fairly similar. The difference is methodological: previous studies typically examined only end-stage schizonts (collected via egress-arresting drugs), whereas we chose to avoid this additional perturbation and examined the whole progress of S-phase at a population level, agnostically. Therefore, we took all the cells at timepoint x, rather than just the ones that looked like end-stage schizonts (a rather subjective judgement at least unless a drug arrest is used - for example, Reilly et al. purified via MACs and then visually selected ‘segmenters’, yielding strain-specific medians of 15-18, ranges of 8-26, whereas Simon *et al.* drug-arrested their schizonts prior to egress, yielding higher strain-specific medians of 18-20, ranges 11-30).

Since S-phase was imperfectly synchronous across the population (which is part of the biology we set out to capture), some smaller cells still remained at all late timepoints. We did not exclude these, whereas the previous references would likely have done so. Even when a late timepoint was dominated by mature schizonts, these lagging cells would therefore bring down the median number of nuclei. We have added detail to the text (lines 142-146) to clearly distinguish our methodology from previous papers.

The authors claim that “The length of S-phase is proportional to the overall length of erythrocytic schizogony”. I think their data shown in Fig. 2 is informative overall and underpins that schizogony takes about 30% of the cycle. This, however, shouldn’t be mistaken for a correlation between total cell cycle length and schizogony at the single cell level and clarified accordingly.

We have clarified that timeframes on the population level do not necessarily correlate cell-to-cell with the single-cell level (line 152).

The authors claim that centrin signal temporarily disappears from a majority of nuclei and suggest this represents a centriolar plaque disassembly. To my knowledge temporary centrosome disassembly (down to centrin) during a division phase is not described in any eukaryote. For Plasmodium specifically multiple studies investigating centrin don’t report any similar findings although their imaging data quality is good (Arnot et al. 2011, Roques et al. 2019, …). Particularly the Simon et al. study referenced by the authors, as far as I understand does only refer to 50% of mononucleated nuclei lacking a centrin signal while in multinucleated stages all nuclei carry one or two centrin foci. Finally, the signal-to-noise ratio shown in Fig. 4B casts significant doubt on the validity of this rather bold claim.

See above – we have revised to remove this misinterpretation of the Simon *et al* data, and to qualify our own findings in the light of these data, which used a custom non-commercial centrin antibody (line 224).

Most importantly, the authors fail to demonstrate that cell cycle progression was “not overtly affected” (line 117) by their labeling. This is particularly critical in the light of what the last author has shown in Merrick et al. 2015 where TK expressing parasites displayed a high sensitivity to BrdU (and possibly EdU?) and a severe growth phenotype at significantly lower concentrations than used in this study (IC50 of 219nM measured for a 1 hour pulse – against - 200µM pulse for 30min used here). Unfortunately, this puts into question the interpretation of a big proportion of the data and the authors have done very little to alleviate this concern. BrdU toxicity could for example be the cause for the replication gaps observed for a subpopulation of nuclei in Fig. 6. This is probably even more critical for the observations made in Fig. 7 that nuclei seem to pause replication for extended periods of time.

We agree that this is a fundamental limitation of this method: modified nucleotides are (for unknown reasons) toxic to *Plasmodium*. However, all our studies suggest that this is not the case until the final stage of schizogony: as shown in our original paper Merrick, Malar J 2015. BrdU-labelled nuclei seem to generate merozoites that do not effectively reinvade, but the cell cycle appears normal up until then. Our strategy here - brief pulse-label experiments earlier in schizogony - is therefore valid, reflecting parasite biology accurately.

In support of this: A) in the figure-2 timecourses we simply labelled cells for the final 30mins prior to fixation, meaning the cell cycle could not be affected up until this modified nucleotide hit the cells. The overall results from these timecourses (numbers of progeny, overall time of S-phase, etc.) were very similar to those from the second set of timecourses, where the first pulse-label went in some hours before fixation and could theoretically perturb S-phase. Evidently, therefore, the second timecourses were not affected by any toxicity from carrying labelled DNA for some hours before fixation. B) key parameters, such as the time taken by a nucleus to replicate, are very similar in our data and in data from the Ganter lab (*Science Advances* March 30^th^ 2022), using an orthogonal method of live-imaging GFP-PCNA. His method brings its own potential issues of photo-toxicity, which must be mitigated, but it is completely independent of any BrdU issues. These points are elucidated in the discussion text (lines 413-415) to provide reassurance that BrdU toxicity is not an issue in the protocols we used.

Reviewer #2: General
My main concern is that it appears that for all data presented in the study, there is only a single biological replicate, in particular for assays run with P. falciparum. Whether there are multiple biological replicates or only one should be specified for every experiment in the figure legend. Suppose there is only a single biological replicate. In that case, the authors should provide extensive justification for why this is the case, and this should be reflected by significantly dampening the corresponding conclusions.

See above re. the second set of timecourses that we presented in the original Figure 6, which acted as replicates of the method, while also supplying additional novel data. However, in the revision we have also now made ‘exact’ replicates of several timecourses (Supp Fig 3 and 7), showing again that the first set of timecourses were largely reproducible.

The authors note the difference in observed lifecycle length for P. knowlesi based on different batches of donor blood and in different P. falciparum 3D7 parasite line ranging from ~39 hours to the ~48 hours reported in this study (Wockner et al., 2020 (PMID: 31679015); Duffy and Avery, 2017 (PMID: 28738214). Considering the potential influence of lifecycle length on the estimation of the length of S-phase.

We have clarified in the text that different strains do have slightly different lifecycle lengths, and that this might slight affect our ‘30%’ estimate for S-phase (line 152-3).

In line 184, the authors rigorously and rightfully used the term DAPI-stained area instead of nucleus. The resolution is calculated by the following formula Resolution=1.22 (λ/(NAobj + NAcond)), if we assume that the authors used an objective with a high numeric aperture or NA (1.45) and a condenser of 0.55, the resolution for the DAPI channel (405 nm) is as best 247.5 nm for the lateral resolution (resolution in the focal plane). Therefore in this study, the resolution is four times bigger than an individual nucleus (200nm at best versus 50 nm); consequently, the authors cannot identify single nuclei in a multinucleated cell and must revise their results statements.

We accept that light microscopy has its limitations and that the apparent DAPI stained area may not perfectly represent the ‘real’ size of a nucleus. However, we are not sure about the reviewer’s suggestion that a single nucleus is only 50nm in size(?) As shown in EM studies, which probably remain the gold standard (e.g. Rudlaff *et al.,* see below), a nucleus even at its most condensed stage in a late schizont is generally at least 1µm wide (and often ~2µm in earlier stages). Therefore, the resolution of light microscopy is adequate for these organelles.

The data from the referenced Simon et al. study are inappropriately referenced to suggest that detection of centrin in ~50% of nuclei is consistent with previous literature. This was only the case in mononucleated parasites with a hemispindle, with the following statement made in that article: “Consistent with the late appearance of PfCentrin1-GFP in our live-cell imaging data, only 24 out of 52 analyzed hemispindles in mononucleated cells were associated with an endogenous centrin signal, whereas in later stages, after the first division, every nucleus was accompanied by one or two centrin foci”. Presumably, from 33-47hpi in P. falciparum and 23-33hpi in P. knowlesi, you have relatively few mononucleated parasites. Therefore the ~50% of nuclei lacking centrin staining likely represents a vast number of false negatives.

Agreed – see the response on this issue above. Our work was all performed prior to the publication from Simon *et al.*, but after a recent discussion with these authors, we believe that under-detection of centrin foci must be primarily due to our use of a commercial antibody. After encountering similar limitations, they chose to raise their own antibody, together with using a *Plasmodium* GFP-tagged line. We have revised our text (line 224) and acknowledging that improved centrin-detection has now been achieved, we have moved our own data on centrin to supplementary.

Finally, the anti-centrin antibody labels only one protein present at the cytosolic face of the MTOC, which is a large multiprotein structure embedded in the nuclear envelope for which we know very few components. The authors should address this and consider this when making conclusions about centrin and MTOC.

Agreed – our discussion already explicitly makes the point that centrin is only one component of a complex structure, and since more data have come out about this structure, this is very much confirmed.

Results
Line 115: “We confirmed that this permitted the labelling of DNA replication with modified nucleotides (bromo/chloro/iodo/ethyl-deoxyuridine (BrdU/CldU/IdU/EdU)), and that parasite behaviour in terms of fitness and cell-cycle timing was not overtly disturbed". Data is not shown or unclear where the authors presented it in the manuscript.

We have added a supplementary figure (S1) showing that in the newly-generated *P. knowlesi* TK-expressing line, versus the parent line, DNA replicates at a similar rate through S-phase (as measured by accumulation of SYBR-green stained DNA). We also note that a) the overall cell cycle times observed here in the TK-line are similar to those previously reported by the Moon lab and others in the parent line (~28h, subject to some variability in human erythrocyte batches), and that b) merozoite numbers up to a max of 16 are consistent with counts by Simon & Guizetti 2021 (doi: 10.3389/fcimb.2021.658616).

Line 133-135: the number of nuclei detected at the latest timepoints for schizonts are drastically lower than previously published numbers of merozoites per schizont, at least for P. falciparum where this has been well characterized (Garg et al., 2015 (PMID: 26702305); (Rudlaff et al., (PMID: 32511279)). The authors should elaborate on why this is the case, be it an issue of resolution, parasite maturity, or something else, and clearly state this when making conclusions based on the number of nuclei per cell.

As above re Reviewer 1 – it is likely an issue of parasite maturity within the population and we have now discussed this further in text (lines 142-46).

Line 139: “…the onset of S-phase for the majority of cells was quite synchronous (Figure 2C, D) particularly in P. knowlesi, where ~60% of cells commenced replication within a single hour at 22-23hpi.” This doesn’t seem to be supported by the data in Figure 2C & D. According to the methods, these cultures are synchronized to within 1 hour of each other, yet there is a 4 hour difference between when the first parasites begin to replicate (22hpi) and when the greatest number of parasites are replicating (26hpi). This could be interpreted as up to 4 hours of replication asynchrony within a population that should be within 1 hour of age.

Unfortunately, synchronising to a single-hour window at 0hpi never, in our hands, results in the maintenance of strict 1h window as the cell cycle progresses. The parasites drift during ring-stage (possibly because all erythrocytes aren’t identical and some invade ‘better’ host cells than others). Our experiments highlight this fact in a way that other population-based approaches may not. Nevertheless, our statement is correct that 60% of the Pk population did start labelling with BrdU within a single hour (22-23hpi). The remaining parasites were the putative ‘drifters’ that lost more than 1h as they progressed through ring stage, and hence entered S-phase in the subsequent hours.

Line 145: “Numbers of nuclei stopped increasing at 46-47hpi, making 15 hours the total S-phase period across the population.” It seems in Figure 2C that ~80% of cells are EdU positive at 47 hours, indicating that at 47 hours, 80% of cells are still replicating and could very well increase in DNA content resulting in the formation of new nuclei past the 47 h.p.i. Considering that 80% of parasites were still replicating at this time point, it is difficult to see how it can be used to conclude the length of the total S-phase. The measure of the total content of DNA would be a more appropriate measure than many nuclei, which again cannot be resolved with the microscopy technique presented in the study without treating the parasite with E64, which would allow to increase in the physical distance between nuclei and allow their counting by light microscopy. Therefore the data presented in Figure 2, panels A, B, C, F, G, and H must be revised and interpreted accordingly.
Line 153-154: “P. falciparum showed substantial reinvasion by 48hpi (Supp Figure 2), and so has a similar window of ~2h between the end of S-phase and reinvasion.” Supp Figure 2 doesn’t appear to have any data regarding EdU labelling, and so can’t be used to support the claim that S-phase has ended.

We agree that it is not ideal that the first timecourse, shown in Fig 2C, stopped at 47hpi, before significant reinvasion had occurred (by contrast, the first Pk timecourse was followed through to reinvasion). However, the second timecourse in P. falciparum, which was shown in Fig 6 and in Supp fig 2, did go through to 48hpi and showed substantial reinvasion at this point. Similarly, the new ‘exact replicate’ timecourse that we have now generated in *P. falciparum* (new supp Fig 3) was followed through to revinvasion at 48h. Therefore, we believe it is correct that this was genuinely the end of S-phase.

Line 186-189 “…particularly notable in P. falciparum, was a subset of nuclei with homogenous but fainter staining, suggesting that DNA had replicated much less rapidly than in the common pattern: this appeared mostly in late-stage cells completing their presumptive final round of replication (Figure 3B, see P. falciparum at 44-45hpi, P. knowlesi at 30hpi, and quantification in Figure 3C, D)” There are many reasons why fluorescence intensity may differ between different cells, and it is unclear why DNA replicating more rapidly would be the reason for this difference.

We agree that other explanations are possible for reduced brightness in the final-round nuclei. However, the simple idea that less DNA was replicated within 30mins – so less EdU was incorporated for fluorescent detection – seems to us the most obvious explanation. We do already suggest in the text (line 457) that instead of replication being fundamentally slower, repair synthesis may be occurring at a low level in all areas of all nuclei, but we consider this less likely. Theoretically, it could also be that specifically in the final round, modified nucleotides enter the pool much less efficiently, resulting in less EdU labelling per the same amount of DNA replication – but again, we have no evidence or biological explanation for this alternative idea.

Also, your study and others suggest that DNA replication occurs faster at the later stages .of schizogony, which contrasts with the idea that DNA had replicated less rapidly in these cells.

Indeed, the overall conclusion (both from our work and that of the Ganter lab) is that replicative rounds become faster over the course of schizogony – and therefore something quite distinct must happen with the overall slowdown that we surmise to occur in the final round. Possibly, as we suggest in the text, they are all slowing and somewhat ‘synchronising’ prior to cytokinesis (line 455-457).

Line 210-212: “In both species there was some evidence that nuclei with centrin foci anti-correlated with actively replicating, centrin-negative nuclei see for example 42hpi in Figure 4D). These observations point to a temporal separation between the start of DNA replication and the start of centriolar plaque elaboration and duplication”. Considering the previously mentioned likely high false negative rate, and a lack of statistical tests showing this correlation, the data presented in Figure 4 is not sufficiently robust to support these claims. Additionally, it is unclear how you can infer temporal separation between DNA replication and MTOC without live-cell microscopy.

Although we believe that such temporal separation can at least be inferred without live-cell work – this being the advantage of assessing large numbers of fixed cells over a large number of close-spaced timepoints – we do agree that this is difficult to statistically-test in a stringent way. Therefore, since we cannot support it statistically, we have removed this idea from the text.

Line 249: “Therefore, replicative rounds became progressively faster as ploidy increased” In my understanding figure 5 supports the conclusion that DNA replication is asynchronous in both Plasmodium species and that is all. And because the experimental design does not allow live imaging therefore authors cannot assess the rate of round of DNA replication like it has been done in the preprint of Klaus et al prublished in 2021.

We believe that Figure 5 does give information about the length of S-phase. It is complementary to the live-imaging data from Klaus *et al.* (or so we have concluded via extensive discussion with these authors) because it uses an entirely different method and yields a similar conclusion. They took a series of snapshots of live cells and inferred that a nucleus was continuously replicating as long as it contained GFP-PCNA. We took a single snapshot of many cells labelled with two successive labels (EdU and BrdU) and inferred that a nucleus containing both labels had replicated at least from the end of the EdU pulse to the beginning of the BrdU pulse.

Discussion
Line 367: "The growing anabolic challenge may, however, be one reason why the gaps between replicative rounds increased as schizogony progressed. It may also account for why some nuclei appeared to arrest for much longer than average". The authors assess DNA replication in this study and not DNA segregation and karyokinesis. The authors call the arrested nucleus what might be an anaphasic nucleus when the two replicated interpolar microtubules connect DNA masses within the same nuclear envelope upon nuclear fission. It is unknown how long this step takes during plasmodium mitosis and whether or non this process is shorter in time as schizogony progresses.

Agreed – ‘arrested nuclei’ could be arrested either in anaphase OR before replication is complete: we cannot distinguish these and have made this point clearer in the text (line 389).

Line 405: "centriolar plaques are disassembled with each replicative round". This relates to the previous comment regarding centrin not being an appropriate marker for the entire MTOC and the high false-negative rate. Supplementary Video 2 of the Simon et al. study shows first the first ~2-3 rounds of mitosis, where intranuclear microtubules are always associated with at least one centrin focus. Additionally, in another study where MTOC of P. falciparum was observed (using NHS ester instead of centrin), an MTOC was observed for every nucleus for every image included in the study (Liffner & Absalon, 2021 (PMID: 34835432), see supplementary videos).

Agreed – the MTOC field is fast evolving and we produced these data before either the work of Simon *et al.* or Liffner e*t al.* were published. We have modified accordingly.

Figures
While the median is often reported, there are no error bars presented on any of the data in the study. Therefore, the authors should include and define error bars for all data where multiple data points are reported.

We cannot find any figures where providing error bars would be appropriate. Wherever a single dataset consisting of a long series of timepoints is reported (each timepoint having a median), such as in Figure 2, it would not be possible to generate error bars, because each timepoint is a distinct single dataset. When these timecourses were repeated later in the paper, for example with the addition of double-labelling protocols, it would still not be appropriate to combine the two datasets and make error bars (i.e. ‘range bars’) because these are all scatter-plot representations. Therefore, we have shown all replicates as separate supplementary figures instead. It *would* be possible to represent the data in a different way – e.g, box plots with mean and interquartile range – but such representation would hide the full raw data: we intentionally chose scatter plots because every cell or nucleus is represented and the raw data are therefore visible.

Many of the microscopy images presented in this study lack scale bars or only one image in an entire panel of figures will have a scale bar. Therefore, either all images should include defined scale bars. If the imaged area is identical for all images in a particular panel, this should be stated in the figure legend that the same scale bar can be applied to all images.

No figure lacks a scale bar entirely, but we have put only one bar on each figure, to reduce clutter on these very busy figures. We have added to the figure legends the fact that the same scale bar applies to all panels within a figure.

For Figures 3a and 4b, it is currently unclear which organism these images represent. Therefore, the representative images should include examples of EdU and centrin staining classifications for both P. falciparum and P. knowlesi.

We have clarified that the representative images are of *P. falciparum*, but are equally representative of nuclei from either species.

Methods
Based on the synchronization protocol listed in the methods, the authors seem to purify viable ring-stage parasites using Nycodenz. However, the authors do not reference a previous study establishing this protocol. If this protocol was developed in this study, the authors should provide validation data that it purifies ring-stage parasites or provide a reference if developed elsewhere.

We have added a reference to the protocol, published by Mohring et al., 2020.

The numerical aperture for the objective lens used in this study should be stated.

Added.

Reviewer #3: Major Points
The experimental design is laid out in Fig 1, and numbers of cells examined are mentioned in several figures but it is not clear what biological repeats have been carried out. Are cells examined all from the same cultures on same day? Or imaged from different repeat experiments. I would expect some parameters measured to vary depending on how “happy” a given culture was, and so data from independent cultures is vital. Could the authors clarify how biological repeats were undertaken, clearly stating this in each figure.

All cells in each timecourse are from a single culture followed continuously throughout the entire timecourse. For the second set of timecourses where the same time period was followed but with additional labelling complexity, we used separate cultures of Pf and Pk, on different days. The additional new replicates that we have now added as supp Fig 3, replicating the data in Fig 2, were also separate cultures, each followed through a complete timecourse. Indeed, some parameters did vary, depending we believe primarily on blood batch, hence we observed that the Pk cell cycle was longer than usual in one timecourse. In our hands, Pk seems to be particularly sensitive to the age of the human erythrocyte batch. We had noted this in the original text, but we have now expanded upon it, and clarified the status of the replicates more clearly in the text (lines 165-175).

**Part III – Minor Issues: Editorial and Data Presentation Modifications**
Please use this section for editorial suggestions as well as relatively minor modifications of existing data that would enhance clarity.

Reviewer #1: Generally, I notice a “disconnect” between the findings claimed in the abstract, the sub-titles of the results and figures, and the conclusion made at the end of each sub-chapter of the results. This leads to difficulties in clearly grasping the main findings of the study. Associating the schematics of the experimental setup directly with the corresponding figure would be very helpful to the reader to easier understand the complex data.

We have tried to make this more clear in the revision, and have added schematics together with the associated figures.

The category “fully stained, less bright” used in Fig. 3 bears some risk of not being clearly separatable from the background. Possibly a more objective criterion could be applied here. Maybe it is also helpful to stratify the integrated densities shown in Fig. 3D by the categories shown in Fig. 3C.

We are aware that categorising staining patterns is inherently subjective; hence we tried to be quantitative wherever possible: for example, ‘part-stained, less than half the DAPI-stained area’ vz ‘part-stained, more than half the DAPI area’. Similarly, to be quantitative about the less-bright pattern, we quantified the integrated density of EdU staining (Fig 3D). In a new supp figure (S4), we have now stratified the integrated densities shown in Fig. 3D by the categories shown in Fig. 3C, as suggested.

For Fig. 5 it would be good to show a simultaneous labeling with BrdU and EdU as a positive control.

We have added this in the supplementary data (supp Figure 6).

The interpretation of the data collected in Fig. 6 could benefit from a more detailed explanation or visualization. Further, the authors could improve on their argumentation how their pulse labeling protocols distinguishes between replication length, replication gaps, and continuously, or not at all replicating nuclei in a multinucleated population.

We struggled to find a better visualisation in Figure 6 (now 5), but have added more schematics associated with each figure.

The authors use the term “syncytial mode of replication” in the abstract. The word syncytium is, however, clearly defined as a multinucleated cell resulting from cell-cell fusion not from multiple nuclear divisions without cytokinesis (Daubenmire, 1936, Science and Wikipedia). Although I have noticed this term see casually applied for Plasmodium spp. I want to strongly advice against this usage.

We have removed this term.

Reviewer #2: Introduction
Line 47: “The malaria parasite Plasmodium”. This should be revised to say the genus of malaria parasites.

Edited.

Line 95: the following is posed as an unanswered question “Do all successive rounds of nuclear replication take the same time?” This question has largely been addressed by the Simon et al., and Klaus et al., studies cited elsewhere in this paper.

We appreciate that data are fast appearing on this issue: neither of the cited papers was available when this work was written up, and still on preprint at the time of our submission. We believe that all these studies, including our own, provide complementary information – for example, the Klaus *et al*. study cannot look beyond the first couple of replications, and provides only a theoretical model for the rest of schizogony. Now that this paper is published in its final version (March 30^th^ 2022) we have added to our discussion comparing our work with this study.

I personally appreciate when authors conclude the introduction section by summarizing the major findings of the study, which is missing here.

We are aware that the paper is already lengthy, so we avoided making the introduction any longer by previewing results upfront.

The following statements should be qualified with references:
Line: 55 “Parasitaemia (Parasitemia) in human malaria can vary greatly, partly because the human-infective Plasmodium species (P. falciparum, P. vivax, P. malariae, P. knowlesi, and P. ovale wallikeri & curtisi) differ in their preferences for human erythrocytes.”
Line 58: “…P. vivax is restricted to invading scares reticulocytes, P. malariae primarily invades older erythrocytes, and P. falciparum invades cells of all…”.
Line 61: “There are also other inter-species differences in the process of schizogony. Its duration varies from ~72 hours in P. malariae to ~28 hours in P. knowlesi and the number of merozoites produced per schizont varies from as many as ~30 in P. falciparum to ~15 in P. knowlesi.”
Line 68: “…P. vivax and P. knowlesi have A/T contents of only 60 and 61%.”
Line 71: “These differ in parameters such as genome content, cell cycle period and merozoite number.”
Line 81: “…the first round of DNA replication begins more than halfway through the cell cycle (which is theoretically 48h, actually ~42-50h in different stains and culture systems).”
Line 85: “Duplication of the centriolar plaque (the Plasmodium centriole-equivalent) has been proposed to initiate each replication.”
Line 98: “At the single-molecule level, our recent work defined the average speed of replication forks and spacing of replication origins in P. falciparum schizogony at 1.2 kb/min and 65kb between origins – broadly similar to parameters in other eukaryotic cells. These averages changed by ~30% over the course of schizogony, with the fastest fork movement and most widely-spaced origins occurring early on.”
Line 102: “The opposite pattern occurs in human cells, where replication speed is usually limited by cellular nucleotide pools and becomes fastest towards the end of S-phase as nucleotide production peaks.”

Several of these are extracts from a whole paragraph for which the reference was given only once at the end. However, many of the statements listed here have now been newly referenced or re-referenced in the revision, as requested.

Results
In the results, it is stated that “…nuclei with the ‘partial’ replication patterns made up 20-30% of the total in P. falciparum at all timepoints, but fewer than 10% of the total in P. knowlesi (Figure 3C)” At the final time point, however, it seems as if the partial replication pattern takes up approximately 30%.

Accepted: we have now noted the exception of this single point.

Considering the resolution of the microscopy used in this study and that images were not acquired in 3 dimensions, the authors should consider simplifying the two centrin foci classifications in Figure 4. In 3 dimensions, two centrin foci could be ‘opposite’ to each other but have moved up or down relative to how the viewer sees the image rendered in 2D. Therefore these would appear next to each other but, in reality, be opposite.

Accepted: however, when we imaged a selection of cells with confocal versus widefield microscopy (see added supp data in S2), we did not actually find such an instance of misinterpretation.

The Simon et al. study referenced in the paper quantifies the time P. falciparum nuclei spend with a single or double centrin focus, beginning at about 150 minutes for the first mitosis and continuing at around 100 minutes for subsequent rounds. This observation should be mentioned when discussing the length of time a nucleus has two centrin foci.

Obviated by the reduction of the text in this section.

The results state that “… nuclei with centrin foci were never seen past the stage of 8-9 nuclei in P. knowlesi (Supp Figure 3).” Despite this, in Supp Figure 3, there are one centrin data points for cells with 10, 11,12, and 15 nuclei.

This meant ‘nuclei with two centrin foci’ – the typo is corrected in the revison

Treatment of cells with BrdU and EdU induces some DNA damage. For nuclei that have been arrested well before other nuclei in the same cell, the authors should discuss the possibility that BrdU or EdU treatment has caused irreparable double-stranded breaks in these nuclei.

We have made a note in the revision (line 391). We did minimise the risk of photo-damage by keeping cells exposed to halogenated nucleotides in the dark at all possible times.

The authors state that the DNA spreading technique used in this study provides a non-uniform stretching factor but then uses a uniform DNA stretching factor determined previously. Could the authors please elaborate on how it is appropriate to apply this stretching factor when the method used provides non-uniform stretching?

The stretching factor that we used was determined as an average, in this same (similarly non-uniform) system using human cells. It is perhaps not entirely appropriate, because noone has previously attempted DNA spreading with haemozoin-rich *Plasmodium* cells, which could plausibly differ from human cells in their viscosity etc. However, we simply do not have any better figures to use. We have made a note to this effect in the text (line 347). In this work, the comparison between Pf and Pk is fortunately the primary point, rather than absolute values.

Based on the asynchrony in mitosis, it seems improbable that 100% of mononucleated cells would be replicating over 45 minutes, but this is shown in Figure 5D. Is it possible that this could be the product of nucleotide pre-loading? If so, the authors should discuss this.

Fig 5D does not show that all nuclei were replicating for a full 45mins, only that they were replicating from at least the end of the first (30min) pulse to the start of the second (15min) pulse.

Figures
Having all the experimental schematics in Figure 1 before the authors explain what they will be used for seems confusing. It may be more intuitive to include each schematic in the figure/panels of the corresponding assay.

Addressed above: altered in the revision.

It is difficult to get a sense of the spread of the data in graphs 2a,b,e-h; 3d; 5b,d and 6b,d. The authors should consider presenting some of these data as violin plots, which could help to display this spread better.

We have considered this, but prefer the completeness of showing all the raw data.

There is no explanation for what the dotted circle is in Figures 3a and 4b. The authors should detail this in the figure legends.

We have made a note in the revised figure legend – it simply highlights the nucleus to focus on.

Based on their use of cyan and yellow for most of their microscopy, it seems the authors are aware of the difficulties of differentiating red, green, and blue for color-blind readers, and this is very much appreciated. However, it should be noted that the yellow and red used to overlay BrdU and EdU are not discernible for multiple types of color-blindness. Therefore, if color-blind friendliness is important to the authors, they should consider changing the red of EdU to magenta or white.

We have altered this in the revision.

In the results, the authors imply that there were 100 cells analyzed at each time point in Figure 3, but there seems to be far less than 100 data points for many of the time points in Figure 3D. Therefore, the number of cells should be clarified in the figure legend.

Clarified in the legend (in timepoints where many had entirely ceased replicating, there was no signal to quantify).

In Figures 4B & D, the second row of graphs seemed confusing. The authors should describe these more thoroughly in the figure legend.

Fig 4B (now moved to supp) is not graphs. We wonder if this might refer to a different figure, but are unsure about which.

Reviewer #3: Minor points
L62 Although cultured P. knowlesi lines can take 28 hours, this is very likely an artifact of culture (in the same way most cultured Pf are in fact shorter than 48h). In this context it would be worth stating it is ~24 hours.

Noted accordingly in the revision.

In a separate but related point, whilst the A1-H.1 has previously been noted to have a lifecycle of ~28hr, it appears to be much longer in these experiments. Can the authors mention this at an appropriate point and suggest a reason? I can’t see anything obvious, but it would be interesting to hear the authors thoughts.

We find that the cycle is occasionally markedly longer in particular blood batches (possibly the parasites do not do well in old erythrocytes) - we have made a note to this effect in the revision.

L71 P. cynomologi can also be cultured, albeit in macaque cells. Changing this to state cultured in human red blood cells or among human infective species would be more accurate.

We have altered this in the revision.

L155 Is budding the correct term here? This suggests progeny growing out from a mother cell?

We have removed this in the revision (merozoites do ‘bud’ from a central body, but the term is perhaps misleading).

L164 this is an especially interesting finding. Whilst there is clearly no upper limit to the proportion of dividing nuclei, I am not sure it is quite right to suggest that there are not factors limiting it at the individual cell level. It may be that cells that have 100% replicating nuclei in one round, end up dipping down to 20% due to limitations in the next. It would have been really nice to examine this in the context of limiting nucleotide availability, perhaps limiting hypoxanthine or some other precursors.

We agree – this is one intervention (along with other nutritional limitations, etc.) that we will address in our next study. Our discussion, together with the now-published paper of Ganter & colleagues, discusses the idea of ‘limiting factors’.

L188 and Fig 3 - Figure 3 legend could explain B in more detail. Also the Centrin staining is introduced here but not explained until fig 4 and next results section. Authors could look at order in which these are explained to make its inclusion in Fig 3 clearer. I presume that the Pk 33 hour image is a ring? That is probably not obvious for some readers. Define integrated density in the figure legend for 3D.

Modifications have been made in the figure legend – the final Pk image is confirmed as a ring, integrated density is now defined in the legend (as an ImageJ function taking the sum of the values for fluorescent signal of all pixels in a selection), etc.

Fig 4 – probably artifact of compiling but fig 4 has noticeably lower resolution than other figures.
Resolution will be double checked in the revision

Fig 5 – Although this experiment is really interesting, it took me a really long time to understand the logic involved (probably my failing!). Could a graphic be added to illustrate it or perhaps further explanation? Perhaps adding this to Fig 1 B and C?

A graphic has been added to this figure, as suggested

L320 convert this to a full sentence rather than parenthetical?

Altered in revision.

L443 The Pf and Pk data look quite similar, was the trend towards faster movement in Pk skewed by the two very high speed data points, or did it remain if these were removed? Alter description or not accordingly!

We have checked, and find that the trend remains if the two marked outliers are removed, although it is lessened somewhat, as might be expected.
