# Supplementary material for: DNA replication dynamics during erythrocytic schizogony in the malaria parasites Plasmodium falciparum and Plasmodium knowlesi
Source: PLoS Pathog. 2022 Jun 22;18(6):e1010595. doi: 10.1371/journal.ppat.1010595 (PMC9255763; doi:10.1371/journal.ppat.1010595)
Supplement: Attachment — Submitted filename: Final rebuttal 2.docx [file ppat.1010595.s011.docx]

Dear Professor Blackman,

Thank you for the opportunity to make some final text revisions to this manuscript. We have made the requested revisions and provided a point-by-point response detailing these (in red text below). We hope that the manuscript will now be acceptable for publication.

Sincerely,

Catherine Merrick

***********************

**Editorial comments:**

As you will see, both reviewers appreciate and thank you for the revisions made to this manuscript. However, reviewer #2 remains concerned about your capacity to accurately enumerate individual parasite nuclei based on wide-field epifluorescence microscopic images. Whilst we consider that the overall thrust of the work is compelling, we agree with the reviewer that caution should be exercised in some of the statements made in the manuscript and that some further alterations are warranted. We therefore request that you consider making the following minor changes in a second round of textual revisions (no additional experimental work is required):

1. With regard to supplementary Figure 2 (Figure S2); we agree that the reviewer makes several well-founded criticisms of your interpretation of these images, but we consider that this figure should be retained in the manuscript in order that the data are available to readers. As requested, please provide information on the microscope and deconvolution system used for the confocal microscopic images in Figure S2 panel B.
The figure has been retained and detail added to the Methods section as requested.

2. Please consider making the requested changes to Figure 4, Figure 5 and Figure 6 as requested by the reviewer (Fig 4 B and D: use hour post-invasion instead of nuclei number; Fig 4 C and E, Fig 5 E: use replicating DNA instead of replicating nucleus; Figure 6 B, C, E, and F: use DNA masses instead of nuclei).

These changes have been made where possible, as detailed below.

We would suggest that you make it clear in the figure legends, methods or introductory text that the microscopic methodology used inevitably introduces some uncertainly about actual numbers of nuclei in schizonts.
A note on this has been added to the Methods text under data acquisition and analysis.

3. Throughout, as recommended by the reviewer, please use the term schizogony (once defined) in place of ‘cell-division’. Use the term ‘nuclear multiplication’ instead of ‘replication’ when referring to karyokinesis, and use ‘DNA replication’ when referring to chromatin synthesis.

These text modifications have been made throughout.

**Reviewer's Responses to Questions**

Reviewer #2:

My last and significant disagreement with the authors remains on the ability to distinguish and resolve a single nucleus in multinucleated schizont parasites without a fluorescent nuclear envelope marker and epifluorescence microscopy.
I am pleased the authors referred to one of my favorite FIBSEM papers by Rudlaff et al. The introduction section explains the limitation of standard fluorescent microscopy.
"The attainable resolution of standard fluorescent microscopy is defined by the Abbe diffraction equation (resolution = λ / (2 * NA), where λ is the wavelength of light used–typically 350–700 nm–and NA is the numerical aperture of the objective–typically <1.5). Practically, this limits the amount of information gained from immunofluorescence microscopy to a resolution of ~200 nm, which does not generally allow separation of structures smaller than this size."
In this paper, the authors mentioned that to generate a robust rendering of a single nucleus, and they called "Nuclear connections for 4n and 2n nuclei were at least 100nm wide through at least 5 Z-sections (100nm deep) to ensure that the observations of connected nuclei were robust" see Data analysis section.
In epifluorescence microscopy, the z-resolution is 2 to 3 times the XY resolution. So therefore, even if the resolution is at 200 nm XY, the z resolution would be between 400 nm to 600 nm, higher than the width of a single nucleus, which ranges from 200 to 350 nm.
Therefore epifluorescence images using DNA staining without a fluorescent nuclear envelope marker can reveal an estimate of DNA masses but not individual nuclei.

Regarding support figure 2, I have multiple concerns, and it supports the fact that only DNA masses and not individual nuclei can be visualized. For instance, in Panel A, parasite 1: the two -EdU masses with the individual 1C could be either an individual nucleus or a single nucleus at the mitotic spindle phase, parasite 2, the two +EdU top left look very much like one nucleus at the anaphase stage upon nuclear fission, or they could be the individual nucleus. Again the current experimental setting allows to call for DNA masses or replicating DNA and not for individual nucleus.

To an extent, we agree: the exact stage post-genome-replication cannot be determined by our microscopy (in both cases highlighted by the reviewer, we defined 2 nuclei because there are evidently 2 nuclear masses and ~2x DNA content; however, these genomes may still be in anaphase, for example, and not fully enveloped as genuine ‘individual nuclei’). Since we focussed on DNA replication - i.e. the 1 genome to 2 genomes transition - such a distinction would not alter the conclusions we draw about overall dynamics. Nevertheless, we are willing to change the term ‘nuclei’ to the clumsier but more exact term ‘nuclear masses’ where appropriate. This has been done throughout the manuscript.

The authors compared individual z-stack with the maximum projection obtained by wide-field microscopy based on panel B. Confocal microscopy relies on using a pinhole to collect only the fluorescence at the focal point resulting in increasing the resolution. In other terms, with confocal imaging, the fluorescence from objects outside the focal plane is not detected outside of the focal plane, which is not the case in the figure represented in panel B. Could the authors elaborate on what microscope and deconvolution program they use to run their confocal imaging?

Detail has been added to the methods text under data acquisition and analysis (Zeiss LSM 700, no post-deconvolution to give the ‘fairest’ comparison with non-deconvolved widefield imaging).

In addition, as the author mentions in their manuscript (discussion section): " the centrin foci clearly disappeared in late schizonts after the final round of karyokinesis" therefore, the authors must agree that the third parasite in supplementary figure 2 panel B represents unspecific staining with anti-centrin antibody. I recommend removing supplementary figure 2 for the final manuscript.

The figure has been retained on the advice of the editor, allowing readers to make their own conclusions as to the strengths and weakness of the method used. (It is not certain that the third parasite, which shows only 12 nuclear masses, actually is ‘non-specifically’ stained for centrin. Although a degree of non-focal staining clearly does occur, and tends to worsen with parasite maturity, clear foci are still seen here, and many or all of these nuclei may be readying for a final round of division.)

In conclusion, the authors must make the following modifications to their figures and manuscript, ensuring a robust conclusion of their data without changing the main finding of the manuscript.
• Figure 4 Panel B and D: use hour post-invasion instead of nuclei number

It would make no sense to change this in Figure 4, because the essential distinction in this dataset is whether cells have 1 nucleus or more than 1 nucleus/nuclear-mass (something that is only broadly, not precisely, dependent on hpi). Reallocating the data according to hpi might mean mixing >1n cells in with the 1n population, and thus blurring the essential distinction: whether cells were completing their first genome replication or subsequent ones.

• Figure 4 Panel C and E, figure 5 Panel E: use replicating DNA instead of replicating nucleus

As we have done throughout the manuscript, we have changed the figure legends and results-text (the figure itself does not bear the term ‘replicating nucleus’). These text changes have been made in order to make it clear that we are referring to ‘replicating DNA’ (e.g. by using the term ‘nuclear masses’ in the legend and ‘replicating DNA’ wherever appropriate, rather than ‘replicating nucleus’ or just ‘replicating’).

• Figure 6 Panel B, C, E, and F: us DNA masses instead of nuclei

As above, we have changed the figure 6 legend in the same way, for consistency, to use ‘nuclear masses’ wherever appropriate. We have not changed the labels on the figure, in large part because the term ‘nuclear mass’ is inappropriate for 1-nucleus cells, which defacto have only 1 (enveloped) nucleus until the first division starts.

**Part III – Minor Issues: Editorial and Data Presentation Modifications**
Please use this section for editorial suggestions as well as relatively minor modifications of existing data that would enhance clarity.

Reviewer #2: The terms cell cycle and schizogony have been used interchangeably in the manuscript (for instance, in the discussion section: lines 370, 371). I would recommend the authors to use the term cell division or stick to schizogony not to confuse a non-malaria biologist. In the same line, it would be best to use the term nuclear multiplication instead of replication when the authors refer to karyokinesis and DNA replication when they refer to chromatin synthesis.

These text modifications have been made throughout.
